# On Tapping the Pericardium

**Published:** 1886-04-20

**Authors:** T. G. Stewart

**Affiliations:** M. D., F.R.C.P. Ed., F.R.S.E.; Physician to the Queen in Scotland, Edinburgh, Scotland


### ON TAPPING THE PERICARDIUM.

By Prof. T. Grainger Stewart, M. D., F.R.C.P. Ed., F.R.S.E.
**Physician to the Queen in Scotland, Edinburgh.**

[Dr. Stewart concludes the narrative of a successful case of paracentesis pericardii with the following notice of the published results of the operation :]

It is unnecessary for me to go into the history of it in detail, but I may remind you of its introduction by Romero of Barcelona in 1819, of its being performed by Schuh in one of Skeda's cases, of Trousseau's successful performance and warm advocacy of it, and of the valuable evidence in its favor supplied by Dr. Clifford Allbut, of Leeds. My friend, Dr. Philip, has gone over the literature of the subject for me, and has compared the statistical tables prepared by different writers. Three authorities have collected series of cases, namely, Dr. Hindenlang of Freiburg, Dr. Roberts of Philadelphia, and Dr. Samuel West of London.

Taking West's statistics as basis—Successful, 31 ; unsuccessful, 49—there have to be added from Hindenlang 3 cases, all of which were unsuccessful ; and from Roberts 7 cases, of which 3 were successful and 4 were unsuccessful ; and from other sources (not included in statistics) 7, of which 4 were successful and 3 were unsuccessful. Thus, from all sources we have 97 cases ; successful, 38 ; unsuccessful, 59. With results such as these, it is clear that the operation deserves recognition as justifiable practice in certain cases.

What are indications for its use ?

1. It should be tried wherever life is imperilled by the copiousness of the effusion.

2. It should be tried, even if pericarditis be not in itself dangerous, in any case of considerable pericardial effusion in which the pulse threatens to fall. Whether it be due to inflammatory or degenerative changes in the cardiac muscle, or to general debility from severe or prolonged disease.

It was upon the second rule that I founded my practice in the case under consideration.

What are the best rules for operative procedure ?

1. Exploratory puncture should be made by means of a Wood's syringe or other fine perforated needle, the needle being cautiously introduced at a point where there is absolute dulness and least likelihood of injuring the heart.

2. If serous fluid be found, the fine needle of an aspirator should be introduced at the same point and the fluid drawn off.

3. If purulent fluid be found, either aspiration, or, what is probably better, free incision, should be resorted to and the pus evacuated. The splendid results obtained by the latter plan of treatment by Dr. West and by Professor Rosenstein, of Leyden, must satisfy any one who reads their papers of the value of this method.

4. As to the quantity to be drawn off, opinions are somewhat contradictory. If the fluid be purulent, it is obviously desirable to remove the whole of it as speedily as possible ; if it is serous, I think that this rule does not necessarily hold. While admitting that there is plenty of evidence to show that the pericardium may be emptied or almost emptied without danger to the patient, it appears to me that only a sufficient quantity to give relief should be removed. It is a sound principle that in dealing with vital organs only the minimum amount of interference required should be had recourse to, and especially in cases which threaten failure of pulse is this precaution necessary. It is conceivable that the sudden removal of considerable pressure from the surface of the heart might sometimes lead to a fatal syncope, while the removal of a small quantity of fluid would involve no such danger. You are familiar with the occasional occurrence of syncope when paracentesis of the pleura is being performed, and whatever may be the explanation of this fact, it seems quite as likely to occur in connection with the pericardium. I therefore prefer, as at present advised, to draw off only a small number of ounces, and, if necessary, to repeat the operation, rather than to adopt the method recommended by the majority of authorities, and draw off a large quantity at once.

5. At what point should the puncture be made?

It is not very important what point is selected for puncture, so long as the operation is performed with caution. Obviously, wounding the heart is to be carefully avoided, notwithstanding the fact that it has been wounded, and even penetrated, without seriously bad effect. I should insist upon the puncture being made where there is absolute dulness, and should prefer the fifth interspace as much to the left of the sternum as possible. By such a rule we most avoid risk of injuring the heart.

One other point in connection with this case deserves attention, namely, the character of the fluid which was drawn off. It was markedly bloody, much more so than my experience of post mortems in cases of the kind had led me to expect. One would not be surprised at finding the serum bloody in a case of pericarditis associated with purpura or scorbutus, or even in one associated with malignant disease, but I was not prepared for the appearance of such fluid in this case. Kussmaul, however, has recognised the fact that bloody serum is common in pericarditis, and mentions one case in which, desiring to tap the pericardium, he got a straw-colored serum, and, supposing that he had only reached the pleura, pushed the needle further in and got the characteristic pericarditic fluid. I would, therefore, warn any one who is performing the operation that he need not be startled if he find the fluid of a reddish color.—*Edinburgh Medical Journal, August, 1885.*

